# Influence of Adventist Spirituality on Self-Control and Perceived Stress Among Seventh-Day Adventist Adults in Coastal Peru

**DOI:** 10.3390/healthcare14081078

**Published:** 2026-04-17

**Authors:** Gunther Alonso Huaytalla Sanchez, Juan Marcelo Zanga Céspedes, Zembe Alejandro Saito Roncal, Jacksaint Saintila

**Affiliations:** 1 Facultad de Teología, Universidad Peruana Unión, Lima 15464, Peru; guntherhuaytalla@upeu.edu.pe (G.A.H.S.); marcelozanga@upeu.edu.pe (J.M.Z.C.); 2 Facultad de Ingeniería y Arquitectura, Universidad Peruana Unión, Lima 15464, Peru; alejandrosaito@upeu.edu.pe; 3 Escuela de Posgrado, Facultad de Ciencias de la Salud, Universidad Peruana Unión, Lima 15464, Peru

**Keywords:** Adventist spirituality, religion, self-control, perceived stress, psychological well-being, coping, adults

## Abstract

**Background**: Adventist spirituality has been identified as a relevant psychosocial resource for emotional well-being; however, evidence on its relationship with self-control and perceived stress in specific religious populations remains limited. **Objective**: The aim of this study was to examine the associations between Adventist spirituality, self-control, and perceived stress in a sample of adults belonging to the Seventh-day Adventist Church and residing in coastal regions of Peru. **Methods**: A cross-sectional study was conducted between December 2025 and January 2026 with 506 Seventh-day Adventist adults who completed an online questionnaire. Adventist spirituality was assessed using the Mission Commitment Questionnaire, which captures religious–spiritual commitment through three dimensions: personal devotion, participation, and witnessing. Self-control and perceived stress were measured using standardized scales. Data were analyzed using partial least squares structural equation modeling. **Results**: The constructs showed adequate internal consistency, with Cronbach’s alpha values ranging from 0.875 to 0.951 and composite reliability values ranging from 0.906 to 0.956. Adventist spirituality was positively associated with self-control (β = 0.479, *p* < 0.001) and negatively associated with perceived stress (β = −0.457, *p* < 0.001). Personal devotion showed the strongest contribution to the higher-order spirituality construct. The model explained 22.9% of the variance in self-control and 20.9% of the variance in perceived stress. **Conclusions**: Adventist spirituality, particularly personal devotion, was associated with higher self-control and lower perceived stress. Although the cross-sectional design does not allow causal inference, the findings support the relevance of Adventist spirituality as a psychosocial resource linked to emotional well-being in this religious population and justify future longitudinal studies.

## 1. Introduction

Seventh-day Adventists constitute a Christian denomination characterized by a holistic lifestyle that integrates regular spiritual practices with principles of physical, emotional, and community health [1]. Stress is currently recognized as a major global public health concern, given its negative effects on mental health, emotional functioning, and quality of life. At the same time, difficulties in self-control have become increasingly relevant in modern health contexts, as reduced self-regulation is linked to maladaptive coping, unhealthy behaviors, and poorer psychological adjustment. In this context, identifying psychosocial resources that may strengthen self-control and reduce perceived stress is an important research priority. In the Adventist context, spirituality is marked by an emphasis on the daily practice of faith, which includes personal devotion, regular prayer, systematic study of the Scriptures, and participation in community-based activities [2]. Research has documented that Adventists exhibit lower prevalence of chronic diseases, higher longevity, healthier lifestyles, and psychological patterns associated with lower perceived stress and greater emotional stability [3]. These findings have been attributed, in part, to the interaction between consistent spiritual practices, disciplined habits, and supportive community networks [1,3,4]. For these reasons, the Adventist population represents an ideal context in which to examine the relationship between Adventist spirituality, self-control, and perceived stress.

In the present study, spirituality is not treated as a generic construct detached from religious tradition. Rather, it is approached as a contextualized form of Adventist spirituality expressed within an organized faith community. Although spirituality is often understood as involving meaning, transcendence, and connection with the sacred, and religiosity as involving beliefs, rituals, and institutional practices, these dimensions frequently overlap in religious populations. In this sense, the construct assessed here is better understood as a denominationally grounded religious–spiritual commitment that integrates inward devotional life with participation in church life and witnessing. Accordingly, the interpretation of the findings should be limited to this contextualized expression of spirituality.

Self-control, understood as the ability to regulate impulses, emotions, and behaviors according to internalized values or personal goals, is an essential component of healthy psychological functioning [5]. Spirituality practiced within organized religious communities may foster self-regulatory processes by providing a symbolic, normative, and practical framework that guides disciplined behaviors aligned with spiritual principles [6]. For example, subtle reminders of spiritual beliefs or principles have been shown to temporarily increase self-control capacity [7]; however, long-term benefits in self-regulation are more consistently associated with regular spiritual practices, rituals, and community participation than with fleeting reminders [8].

Moreover, Christian theological traditions emphasize self-control as a spiritual discipline cultivated through intentional practices of self-denial and moral training, highlighting its role as a virtue and a hallmark of spiritual maturity [9]. Empirical studies conducted in Islamic contexts have also found that spiritual intelligence and religious faith positively predict self-regulation, supporting goal-directed behaviors such as memorizing the Qur’an [10].

Spirituality within religious contexts is also associated with lower levels of perceived stress. In Christian populations, including Peruvian samples, higher spiritual well-being has been observed to correlate with lower perceived stress, regardless of demographic factors such as sex [11]. Regular spiritual practices—such as prayer, devotional meditation, and personal reflection—help buffer the adverse psychological effects of stress [12]. Likewise, various spiritual or faith-based interventions have demonstrated effectiveness in reducing anxiety and stress in clinical settings [13,14], supporting their usefulness as complementary components within mental health programs. However, the relationship is neither unidirectional nor universally positive; indeed, studies have reported that experiences of religious conflict or spiritual distress can intensify physiological and emotional stress responses [14,15], suggesting that the impact of spirituality depends on the quality of the spiritual experience and the context in which it occurs.

Despite the growing evidence on the Adventist lifestyle and its impact on physical and mental health, important gaps remain in the literature that justify further research. First, few studies have simultaneously examined Adventist spirituality, self-control, and perceived stress within this population, even though these constructs are closely linked to the spiritual and behavioral principles of the Adventist tradition. The Seventh-day Adventist Church in Peru represents a large faith community; according to the 2025 official Adventist Yearbook, the North Peru Union Mission and the South Peru Union Mission together report 443,815 members [16,17]. Seventh-day Adventism is grounded in a biblically centered worldview and is characterized by observance of the seventh-day Sabbath, an emphasis on holistic health and temperance, and the hope of the Second Coming of Christ [2]. These principles promote disciplined daily habits, spiritual commitment, and meaning-making frameworks that may be relevant to self-control and perceived stress. Moreover, most of the available evidence comes from studies conducted in Anglo-Saxon countries, while Latin American—and particularly Peruvian—research remains limited, highlighting the need to explore these associations within distinct sociocultural and religious contexts.

Additionally, few studies have employed structural models, such as the partial least squares approach (PLS-SEM), to simultaneously analyze the direct and indirect effects among these constructs. This type of modeling is particularly useful for understanding the complexity of the relationships between Adventist spirituality and psychological variables associated with emotional well-being. More importantly, although Adventist spirituality has been consistently linked to lower perceived stress, the potential role of self-control as a key explanatory mechanism in this relationship remains insufficiently explored, particularly in Latin American religious populations. Thus, the present study addresses this gap by examining Adventist spirituality, self-control, and perceived stress within a single analytical framework. Consequently, there is a relevant opportunity to provide empirical evidence that expands current knowledge on how Adventist spirituality relates to self-control and perceived stress in Latin American Adventist populations.

The objective of this study was to analyze the associations between Adventist spirituality, understood as a contextualized religious–spiritual commitment, self-control, and perceived stress in a sample of adults belonging to the Seventh-day Adventist Church in Peru.

## 2. Materials and Methods

### 2.1. Study Type and Design

This was a descriptive cross-sectional study conducted in a sample of Seventh-day Adventist adults.

### 2.2. Participants

The sample was obtained using a non-probabilistic convenience sampling strategy with voluntary participation, as participation was open to all individuals who voluntarily expressed their willingness to take part in the study. A total of 506 adults belonging to the Seventh-day Adventist Church, residing in coastal regions of Peru, and aged 18 years or older completed the online questionnaire. The final sample size was considered adequate for PLS-SEM based on recommended guidelines indicating that the minimum sample should exceed the largest number of structural paths directed at a latent construct. This sampling strategy was appropriate given the exploratory nature of the study and the aim of capturing active members of this religious denomination who were able to read and complete the survey in Spanish.

Participants were informed about the study through announcements shared within Seventh-day Adventist community networks and local congregational communication channels. Individuals who expressed interest received or accessed a Google Forms link that directed them to the electronic questionnaire. On the first page of the form, participants were presented with an informed consent statement and were required to select the option “I agree” before proceeding. The purpose of the study, the voluntary nature of participation, and the right to withdraw at any time were clearly explained. No incentives were offered. Confidentiality and anonymity of all responses were guaranteed. Because recruitment relied on voluntary online participation, the sample may have been influenced by self-selection bias, favoring individuals with greater interest in spirituality, higher digital literacy, or greater willingness to engage in survey-based research.

The questionnaire was administered virtually between December 2025 and January 2026. Participants completed the three instruments described above, along with sociodemographic questions (age, sex, educational level, occupation, marital status). The survey remained available for several weeks, allowing for broad participation within the coastal Adventist community. Only fully completed questionnaires that met the inclusion criteria were retained for analysis.

### 2.3. Instruments

**Adventist spirituality/religious–spiritual commitment (Mission Commitment Questionnaire—CCM).** In this study, the focal construct was not generic spirituality, but a contextualized form of Adventist spirituality operationalized through the Mission Commitment Questionnaire (CCM) [18]. This instrument was developed for use in Adventist settings and captures religious–spiritual commitment through dimensions that reflect both inward and outward expressions of faith. Specifically, it includes personal devotion, active participation in church life, and witnessing or service to others. Thus, the CCM was interpreted as measuring spirituality as lived within the Seventh-day Adventist tradition, rather than spirituality in a universal or non-denominational sense. A total of 36 items were included, grouped into three categories: Active Devotional Life of Discipleship (11 items), Active and Integral Participation in Discipleship (10 items), and Witnessing in Discipleship (15 items). Each item was rated using a five-point frequency scale, with response options categorized as Always, Almost Always, Sometimes, Almost Never, and Never. Instrument reliability was evaluated using Cronbach’s alpha, yielding a coefficient of 0.943, which is considered acceptable for this type of study.

**Self-Control (Self-Control Scale—SCS).** Self-control was measured using the Self-Control Scale developed by Tangney et al. and adapted into Spanish by Macarena del Valle and colleagues. Reliability was assessed using Cronbach’s alpha, yielding a coefficient of 0.84. The scale comprises three factors that reflect a general capacity to regulate behavior and impulses (Non-Reflective Impulse Control), the ability to work effectively and remain committed to personal goals (Self-Discipline), and the capacity to resist temptations or pleasurable activities considered harmful or contrary to long-term objectives (Reflective Impulse Control). The questionnaire includes 28 items grouped into three categories: Non-Reflective Impulse Control (11 items), Self-Discipline (12 items), and Reflective Impulse Control (5 items). Items were rated on a five-point Likert scale with the following response options: Always, Almost Always, Sometimes, Almost Never, and Never.

**Perceived Stress Scale (PSS).** Perceived stress was assessed using the Spanish version of the Perceived Stress Scale developed by Sheldon Cohen and colleagues and adapted into Spanish by Eduardo Remor to evaluate individuals’ reactions when facing stressful situations. Reliability was examined using Cronbach’s alpha, with values ranging from 0.84 to 0.86, indicating good internal consistency. The questionnaire was designed to measure the degree of stress experienced in various life situations “during the past month.” It consists of 14 items intended to assess the extent to which individuals perceive their lives as unpredictable, uncontrollable, or overloaded. Responses were recorded using a five-point Likert scale: Always, Almost Always, Sometimes, Almost Never, and Never.

### 2.4. Ethics Approval and Consent to Participate

Ethical guidelines and standards of conduct in psychological research were respected, and the corresponding tools and procedures were properly followed. Additionally, the research protocol was reviewed and approved by the Research Ethics Committee of the Universidad Peruana Unión (protocol code 2025-CEUPeU-054, approval date 30 December 2025). In addition, informed consent was obtained from the participants. The study was conducted in accordance with the ethical standards and amendments included in the Declaration of Helsinki.

### 2.5. Statistical Analysis

Data were analyzed using IBM SPSS Statistics, version 28. The database was first screened for missing and atypical values. Descriptive statistics, including frequencies, percentages, means, and standard deviations, were calculated to summarize the sociodemographic characteristics of the sample and the main study variables.

The main analytical approach was partial least squares structural equation modeling (PLS-SEM), which was selected because of its predictive orientation and its suitability for examining relationships among latent variables within an exploratory framework. In the specified model, Adventist spirituality was treated as a higher-order latent construct composed of personal devotion, active participation, and witnessing, whereas self-control and perceived stress were modeled as endogenous constructs.

The measurement model was assessed in terms of internal consistency, convergent validity, and discriminant validity. Internal consistency was examined using Cronbach’s alpha and composite reliability (CR), with values above 0.70 considered acceptable. Convergent validity was evaluated using the average variance extracted (AVE), with values of 0.50 or higher considered adequate. When AVE was slightly below 0.50, convergent validity was interpreted in light of composite reliability and the theoretical relevance of retaining the indicators to preserve content validity. Discriminant validity was assessed using the Fornell–Larcker criterion.

The structural model was evaluated primarily through the magnitude and statistical significance of the path coefficients obtained by bootstrapping, as well as through the explanatory power of the endogenous constructs using the coefficient of determination (R^2^). The standardized root mean square residual (SRMR) was reported only as a complementary descriptive indicator and not as the main criterion for model evaluation. Statistical significance was set at *p* < 0.05.

## 3. Results

Before estimating the structural model, the measurement model was evaluated for internal consistency, convergent validity, and discriminant validity. All indicators originally specified in the model were retained. Negative self-control indicators were reverse coded so that higher values reflected greater self-control. As shown in Table 2, the constructs demonstrated adequate internal consistency, with Cronbach’s alpha values ranging from 0.875 to 0.951 and composite reliability values ranging from 0.906 to 0.956. Convergent validity was supported, with AVE values above 0.50 for all constructs except self-control, which showed a slightly lower AVE of 0.481. Although this value falls marginally below the conventional threshold, the self-control construct showed high composite reliability (CR = 0.944) and was therefore retained, although its convergent validity should be interpreted with some caution. Discriminant validity was supported by the Fornell–Larcker criterion, as the square root of the AVE for each construct exceeded its correlations with the remaining latent variables. As complementary descriptive information, the model yielded an SRMR value of 0.056. However, because overall fit indices in PLS-SEM are supplementary rather than definitive, model interpretation was based primarily on construct reliability, convergent validity, discriminant validity, and the significance and explanatory power of the structural paths.

The sample consisted of 506 participants, of whom 54.6% were women. Regarding occupation, approximately half were students (50.2%), followed by salaried workers (26.1%) and independent workers (19.6%). Most participants reported having higher education (81.4%), while only a small proportion had secondary (17.4%) or primary education (1.2%). With respect to marital status, the majority were single (70.0%), followed by married individuals (27.9%). In terms of age, more than half of the participants were between 18 and 29 years old (56.7%), and a smaller percentage were 60 years or older (2.8%) (Table 1).

**Figure 1:**
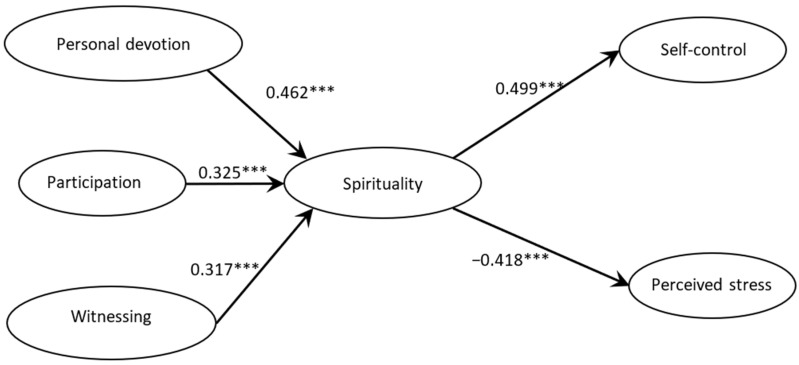
Structural model of the relationships between Adventist spirituality, self-control, and perceived stress. Note. *** *p* < 0.001.

Figure 1 presents the final structural model. The higher-order construct of Adventist spirituality was formed by personal devotion, active participation, and witnessing. As shown in Figure 1 and tables below, Adventist spirituality was positively associated with self-control (β = 0.479, t = 12.25, *p* < 0.001) and negatively associated with perceived stress (β = −0.457, t = −11.52, *p* < 0.001), indicating that higher levels of Adventist spirituality were associated with greater self-control and lower perceived stress.

As shown in Table 2, all constructs exhibited adequate internal consistency and convergent validity. Cronbach’s alpha values were above 0.80, and composite reliability values were above 0.90 for all constructs. AVE values exceeded 0.50 for all constructs except self-control, which showed a slightly lower AVE but high composite reliability, supporting its retention with cautious interpretation.

**Table 2:** Internal consistency and convergent validity of the constructs.

Construct	Cronbach’s Alpha	CR	AVE
Personal devotion	0.918	0.933	0.606
Active participation	0.913	0.928	0.563
Witnessing	0.951	0.956	0.597
Self-control	0.936	0.944	0.481
Perceived stress	0.875	0.906	0.617

Note. CR = Composite reliability; AVE = Average variance extracted.

As shown in Table 3, the structural model revealed significant relationships between the higher-order construct of Adventist spirituality and the two endogenous outcomes. Adventist spirituality was positively associated with self-control (β = 0.479, t = 12.25, *p* < 0.001) and negatively associated with perceived stress (β = −0.457, t = −11.52, *p* < 0.001). Regarding explanatory power, the model explained 22.9% of the variance in self-control and 20.9% of the variance in perceived stress. In addition, the higher-order spirituality construct showed strong contributions from personal devotion (β = 0.400, t = 51.50, *p* < 0.001), active participation (β = 0.358, t = 46.76, *p* < 0.001), and witnessing (β = 0.384, t = 50.90, *p* < 0.001).

**Table 3:** Structural path coefficients and explained variance (R^2^) in the PLS-SEM model.

Path	β	Std. Error	t	*p*	R^2^
PD → SPI	0.400	0.0078	51.50	<0.001	0.985
PAR → SPI	0.358	0.0077	46.76	<0.001	
WIT → SPI	0.384	0.0076	50.90	<0.001	
SPI → SC	0.479	0.0391	12.25	<0.001	0.229
SPI → STR	−0.457	0.0396	−11.52	<0.001	0.209

Note. PD = Personal devotion; PAR = Active participation; WIT = Witnessing; SPI = Adventist spirituality; SC = Self-control; STR = Perceived stress; R^2^ = coefficient of determination.

Age showed consistent effects across the three psychological variables, indicating that older participants tended to report higher levels of Adventist spirituality and self-control, as well as lower levels of perceived stress. Male sex was positively associated with Adventist spirituality, whereas being a salaried worker or an independent worker was associated with lower perceived stress. Educational level and marital status did not show significant associations with the main study variables (Table 4).

## 4. Discussion

The results of this study show that contextualized Adventist spirituality, operationalized as religious–spiritual commitment, was directly and significantly associated with higher levels of self-control among participants. This finding highlights the importance of spiritual life as an internal resource that supports self-regulation, decision-making, and the ability to exert control over everyday impulses and behaviors. In the Seventh-day Adventist context, spiritual life is expressed through structured practices such as regular prayer, Bible study, participation in church services, and observance of the seventh-day Sabbath (from Friday sunset to Saturday sunset), during which individuals typically refrain from work and engage in rest and spiritual activities. These practices promote routine, reflection, and behavioral regulation. In addition, these findings may be better understood within the Peruvian sociocultural context, which tends to value interpersonal connectedness, family bonds, and community participation. In such a context, religious involvement may not only reflect an individual spiritual commitment but also a shared system of norms, mutual support, and collective expectations that reinforce disciplined behaviors and self-regulation. Thus, community-based spiritual life may strengthen individual self-control through both personal conviction and social reinforcement. The positive association between spirituality and self-control observed in this study is consistent with previous evidence indicating that spiritual experience can strengthen self-regulatory and self-control processes.

Several studies have shown that spiritual practices promote states of mindful attention, emotional regulation, and behavioral discipline [10,19,20]. Moreover, spirituality has been linked to a greater ability to resist impulses [21], manage temptations [22], and maintain goal-directed behaviors [23], particularly in contexts involving moral or emotional demands. From a neuropsychological perspective, it has been suggested that consistent spiritual practices may activate brain networks associated with self-awareness and executive control, which could contribute to strengthening self-control [24].

Furthermore, the results showed that Adventist spirituality is significantly associated with lower levels of perceived stress, which aligns with extensive evidence identifying spirituality as a psychological protective factor in the face of stressful situations. Previous studies have documented that spiritual and religious practices can reduce the emotional burden associated with stress [11], possibly through more adaptive coping strategies such as meaning-focused coping, positive reappraisal, and the search for spiritual support. Indeed, recent research also suggests that spirituality fosters feelings of hope [25], purpose [23], and transcendent connectedness [26], factors that buffer both the physiological and emotional response to stress [26,27]. Therefore, the evidence supports the finding that spirituality functions as an emotional regulation mechanism that reduces perceived stress, consistent with the results of the present study.

In addition to the direct effects between the constructs, the study showed that the dimensions of Adventist spirituality exert indirect effects on self-control and perceived stress through the overall spirituality construct. Specifically, personal devotion, participation, and witnessing were indirectly associated with higher self-control and lower perceived stress, suggesting that these practices differentially contribute to the spiritual experience, which, in turn, is linked to the psychological outcomes examined in this study. These findings are consistent with research indicating that both the internal and external components of spirituality may influence well-being through mediating mechanisms such as meaning-making [28] and emotional regulation [29]. However, due to the cross-sectional design of the study, these results reflect only associative patterns, and it is not possible to determine directionality or causal mechanisms between spiritual dimensions and the psychological variables assessed.

A relevant finding of the study was the contribution of the spirituality dimensions to the overall construct, with personal devotion showing the highest loading, followed by participation and witnessing. It is possible that, within this sample, private spiritual practices—such as prayer, reflection, and devotional reading—constitute the most central component of the spiritual experience. This result aligns with previous studies indicating that internal spiritual practices tend to be more stable and more predictive of psychological well-being than communal or expressive practices [30,31]. Nevertheless, given the cross-sectional nature of the study, these findings do not allow for establishing causal directions between spiritual dimensions and psychological outcomes; rather, they reflect associative patterns observed within this specific group of participants. Even so, the stronger contribution of personal devotion to the spirituality construct may suggest that, in similar religious contexts, spirituality experienced in an intimate and daily manner plays a particularly important role in shaping the overall spiritual experience [32].

Regarding demographic variables, the results revealed consistent patterns that help contextualize the association between Adventist spirituality, self-control, and perceived stress. Age was positively associated with Adventist spirituality and self-control, and negatively associated with perceived stress, suggesting that older participants tend to report more consolidated spiritual practices, greater self-regulation, and lower levels of perceived stress. In particular, age showed a significant positive association with self-control and a negative association with perceived stress, indicating that these psychological resources may strengthen over time. This pattern may reflect the accumulation of life experience, improved emotional regulation, and more effective coping strategies developed across the lifespan. This finding aligns with studies reporting that adulthood is often associated with greater stability and emotional well-being [33] as well as spiritual growth [34,35]. Although it is plausible that longer engagement in spiritual practices contributes to these outcomes, the duration or intensity of spiritual practice was not directly assessed in this study and should be considered in future research.

The results also showed that male participants reported higher levels of spirituality compared to women. Although this differs from part of the literature documenting higher levels of religiosity among female populations [36,37], it may reflect particular variations related to the denominational context or the specific composition of the sample. This finding may also be influenced by sociocultural or role-related factors within the religious community that shape different patterns of spiritual expression between men and women. However, this result should be interpreted with caution, as it may not be generalizable to other populations. However, some studies indicate that men report higher levels of certain aspects of spirituality, such as spiritual grandiosity or spiritual self-evaluation, especially among specific groups such as emerging religious leaders [38]. Other research suggests that there are no significant sex differences in religious participation or in the awareness of a relationship with God, indicating that gender differences in spirituality may not be universal or may depend on how spirituality is measured [39].

On the other hand, the finding that salaried and independent workers reported lower levels of perceived stress compared to other occupational groups may indicate that job stability and professional autonomy are associated with more favorable psychological conditions, a pattern also described in research on occupational stress [40]. Indeed, studies conducted in workplace settings suggest that job autonomy generally reduces tension and emotional exhaustion by promoting psychological self-care and resilience, particularly when workload demands are manageable [41,42]. However, among ICU nurses, higher professional autonomy was unexpectedly associated with greater occupational stress, suggesting that increased autonomy may entail greater responsibilities or pressures in high-risk environments [41,42].

### 4.1. Limitations and Future Directions

This study has several limitations. First, its cross-sectional design precludes establishing temporal order or causal relationships among Adventist spirituality, self-control, and perceived stress. Therefore, the observed associations should be interpreted as non-causal.

Second, all variables were collected at a single time point using the same online self-report survey, which may have increased the risk of recall bias, social desirability bias, and common method bias. This issue may be particularly relevant in a religious population, in which participants may be more likely to report attitudes and behaviors that are socially or spiritually desirable. Because no formal statistical assessment of common method bias was conducted, this possibility cannot be ruled out.

Third, the sample consisted exclusively of Seventh-day Adventists from coastal Peru, which limits the generalizability of the findings to other religious traditions, secular populations, and different cultural contexts. In addition, online data collection and voluntary participation may have restricted participation to individuals with greater digital access, higher educational attainment, or stronger interest in spirituality, thereby introducing selection bias.

A further measurement-related limitation is that the AVE for the self-control construct was slightly below the conventional 0.50 threshold, which suggests that its convergent validity should be interpreted cautiously despite its high composite reliability. Finally, although several demographic variables were included, other potentially relevant factors, such as spiritual coping, social support, organizational religiosity, and workplace conditions, were not assessed.

Future studies should use longitudinal or experimental designs to examine temporal relationships more clearly and to explore possible explanatory mechanisms. Research including more diverse religious and non-religious populations would also help determine the extent to which these associations are specific to Adventist spirituality or generalizable to other contexts. In addition, future studies should complement self-report measures with objective indicators and formally assess common method bias using appropriate statistical procedures.

### 4.2. Practical Implications

The findings of this study offer practical implications primarily for faith-based educational, community, and pastoral settings involving Seventh-day Adventist or similar religious populations. In contexts where religious experience and devotional practice are central to daily life, practices such as personal reflection, devotional habits, participatory church activities, and community expressions of faith may be considered as complementary resources to support self-regulation and coping in stressful situations.

These implications should be interpreted with caution because the data were cross-sectional and obtained from a specific religious sample. Accordingly, the present findings should not be directly generalized to secular populations or to religious groups with different theological traditions or spiritual practices. Future studies should examine whether similar associations are observed in other religious communities and should evaluate the effectiveness of faith-sensitive interventions through longitudinal, experimental, or comparative research designs.

## 5. Conclusions

The results of this study show that Adventist spirituality, understood as a contextualized religious–spiritual commitment, was associated with higher levels of self-control and lower levels of perceived stress among Seventh-day Adventist adults from coastal Peru. Additionally, the dimensions of personal devotion, participation, and witnessing contributed differentially to the higher-order construct, with personal devotion emerging as the component with the greatest relative weight.

These findings suggest that, within this specific religious context, spiritual experience—particularly in its private devotional dimension—may play an important role in emotional self-regulation and coping with stressful situations. However, given the cross-sectional design, the observed relationships should be interpreted as associative rather than causal.

Overall, the present study contributes evidence on the relationship between contextualized Adventist spirituality, self-control, and perceived stress in a Peruvian religious population. The interpretation and potential application of these findings should remain limited to comparable religious settings unless supported by future research in more diverse populations.

## Figures and Tables

**Table 1:** Sociodemographic characteristics of the participants (N = 506).

Variable	Category	n	%
**Gender**	Female	276	54.55
	Male	230	45.45
**Occupation**	Homemaker	8	1.58
	Student	254	50.20
	Retired	9	1.78
	Student and worker	4	0.79
	Salaried worker	132	26.09
	Independent worker	99	19.57
**Educational level**	Primary	6	1.19
	Secondary	88	17.39
	Higher education	412	81.42
**Marital status**	Single	354	69.96
	Married	141	27.87
	Divorced	7	1.38
	Widowed	4	0.79
**Age (years)**	18–29	287	56.72
	30–39	85	16.80
	40–49	87	17.19
	50–59	33	6.52
	60 or older	14	2.77

**Table 4:** Effect of demographic variables on levels of Adventist spirituality, self-control, and perceived stress.

Variable	Adventist Spirituality	Self-Control	Perceived Stress
(Intercept)	–0.741	–0.888	1.580
Male (Gender)	0.301 ***	–0.050	–0.103
Age	0.020 ***	0.011 *	–0.012 *
Occupation: Student	0.450	0.112	–0.583
Occupation: Student and Worker	0.615	–0.928	0.172
Occupation: Retired	0.106	0.388	–0.820
Occupation: Salaried Worker	0.261	0.260	–0.735 *
Occupation: Independent Worker	0.173	0.292	–0.746 *
Educational Level: Secondary	–0.245	0.293	–0.453
Educational Level: Higher Education	–0.269	0.420	–0.576
Marital Status: Divorced	–0.109	0.345	0.343
Marital Status: Single	–0.110	–0.013	0.040
Marital Status: Widowed	0.899	0.021	–0.709

Note. * *p* < 0.05; *** *p* < 0.001.

## Data Availability

The dataset generated and analyzed during the current study is not publicly available due to privacy and ethical restrictions related to the protection of participant anonymity. However, the data may be obtained from the corresponding author upon reasonable request and subject to approval by the research team.

## Abbreviations

The following abbreviations are used in this manuscript:

CCM	Mission Commitment Questionnaire
SCS	Self-Control Scale
PSS	Perceived Stress Scale
PLS-SEM	Partial Least Squares Structural Equation Modeling
SPI	Adventist spirituality
SC	Self-Control
STR	Perceived Stress
PD	Personal Devotion
PAR	Active Participation
WIT	Witnessing
SPSS	Statistical Package for the Social Sciences
